# Expression pattern of estroprogestinic receptors in sinonasal inverted papilloma

**DOI:** 10.18632/oncotarget.17161

**Published:** 2017-04-17

**Authors:** Agostino Serra, Rosario Caltabiano, Giacomo Spinato, Salvatore Gallina, Salvatore Caruso, Venerando Rapisarda, Paola Di Mauro, Veronica Castro, Angelo Conti, Luisa Licciardello, Luigi Maiolino, Salvatore Lanzafame, Salvatore Cocuzza

**Affiliations:** ^1^ Department of Medical and Surgical Sciences and Advanced Technologies “G. Ingrassia”, ENT Section, University of Catania, Catania, Italy; ^2^ Department of Medical and Surgical Sciences and Advanced Technologies “G. Ingrassia”, Section of Anatomic Pathology, University of Catania, Catania, Italy; ^3^ ENT Department, Rovigo Provincial Hospital, Rovigo, Italy; ^4^ Department of Experimental Biomedicine and Clinical Neurosciences, Otolaryngology Unit, University of Palermo, Palermo, Italy; ^5^ Department of General Surgery and Medical Surgical Specialties, Gynecological Clinic and Research Group for Sexology, University of Catania, Catania, Italy; ^6^ Department of Clinical and Experimental Medicine, Section of Occupational Medicine, University of Catania, Catania, Italy

**Keywords:** paranasal sinuses, inverted papilloma, human papilloma virus, hormonal receptor expression, immunohistochemistry

## Abstract

Inverted papilloma (IP) is a locally destructive, benign neoplasm of the nose and paranasal sinuses with a high tendency for recurrence, a significant potential for malignancy, and an etiology that today is still uncertain. The expression of hormonal receptors in neoplastic tissues has been the focus of intensive research for its potential diagnostic, prognostic, and therapeutic significance. The aim of this study was to assess the potential estroprogestinic receptor expression in patients undergoing sinus surgery for IP. A retrospective study was carried out, on surgical specimens of 73 patients who underwent endoscopic sinus surgery for first manifestation of sinonasal IP (primitive IP group) and in 21 subjects who had developed a recurrence (relapsed IP group). The results of the immunohistochemical analysis of the first group showed the absence of receptor expression for PGR in all cases analyzed and the presence of a low positivity for ER in 11 cases (*P* > 0.082). Similarly, in the second group the results showed a low presence of ER receptors in 3 of the 21 cases (*P* > 0.068), while there was no evidence of PGR receptors in the examined samples. In addition, in 11 of the cases only 3 were considered positive (27.2%) showing a recurrence during follow-up (*P* > 0.068).

Our results suggest that the sinonasal IP is a benign tumor independent of estrogen and progesterone, and the receptors for these hormones are therefore unsuitable as predictors of relapse or possible prognostic indicators and therapeutic targets.

## INTRODUCTION

The inverted papilloma (IP) of the nose and paranasal sinuses is a benign tumor that, today is the focus of research concerning the most recent acquisitions in terms of etiopathogenesis, pre- and post-operative clinical work-up, prognostic factors and different surgical strategies aimed at achieving a more radical exeresis and a lower relapse frequency.

There has been a growing number of interventions on the nasal side wall and a number of technological innovations over the last decade that, after due consideration, have led to targeted surgical approaches and less invasive demolition.

On the etiological side, several studies have provided greater clarity on a topic still relegated to the probable association with the human papilloma virus (HPV), which has never been identified in cell cultures, if not through molecular hybridization techniques, in which the HPV DNA was detected in 73% of the IPs [1], the RNA of HPV types 6 and 11 was found in 89% of sinonasal papillomas concomitant with similar forms in the genital tract [2], and DNA sequences HPV 6b and 11 were isolated by Weber in 76% of cases [3].

On the other hand, especially in the last decade, there has been the identification in many cancers of the head-neck, renal, and breast district of receptors for sex hormones This is thanks to technological innovation in the biological field of a more sophisticated immunohistochemical methodology with greater sensitivity. This expression could therefore be the basis of a hormone-dependence, which could play a central role in prognosis and therapy [4–5]. On this basis, different pharmacological strategies are available to obtain volumetric tumour shrinkage and/or arrest of progression in various benign or malignant tumors including a reduction in time to progression and disease recurrence [6].

The aim of this study was to assess the potential estroprogestinic receptor expression in patients undergoing sinus surgery for IP, to identify new etiopathogenetic factors and/or co-factors useful for prognostic and therapeutic development.

## RESULTS

**Figure 1:**
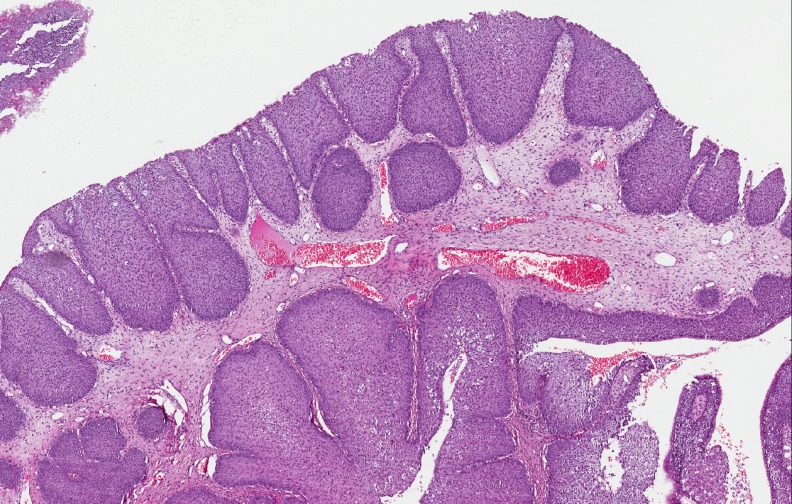
IP characterized by an inverted pattern of growth with cords and nests of epithelium endophytically projecting into the underlying stroma (H&E; 100×)

At the histological examination, the IP showed an inverted pattern of growth with cords and nests of epithelium endophytically projecting into the underlying stroma (Figure 1). At higher magnification, the surface of the IP had from 5 to 30 layers of squamous, ciliated columnar, or intermediate epithelium with interspersed mucus-secreting cells. In all epithelial types, nuclei were generally small and uniform, with darkly staining chromatin and rare nucleoli. Mitoses were few and confined to the lower epithelial levels. Dilated ductal-like structures, lined by multiple layers of epithelium and terminating in smaller cellular nests, were usually observed.

**Figure 2:**
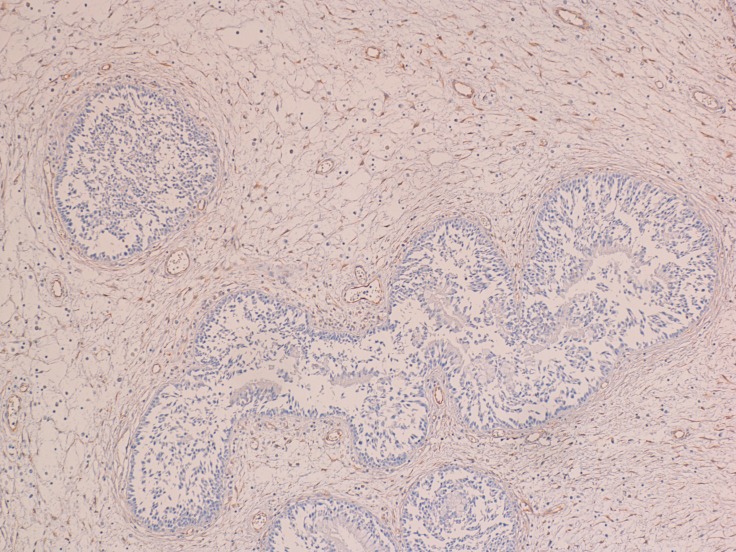
The epithelial cells of primitive IP were negative for progesterone receptor (immunoperoxidase; 100×)

**Figure 3:**
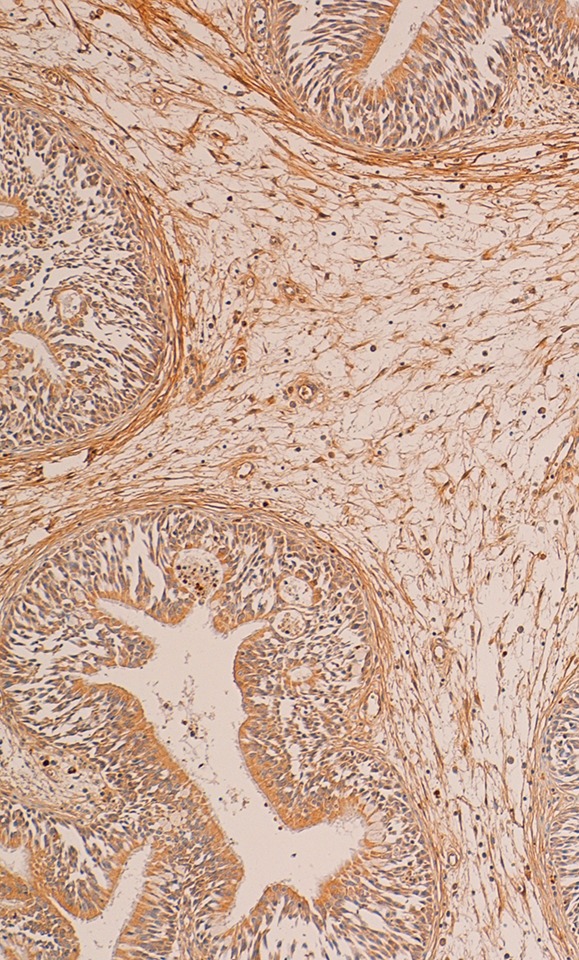
A case of primitive IP with a low positivity for estrogen receptor (immunoperoxidase; 150×)

**Figure 4:**
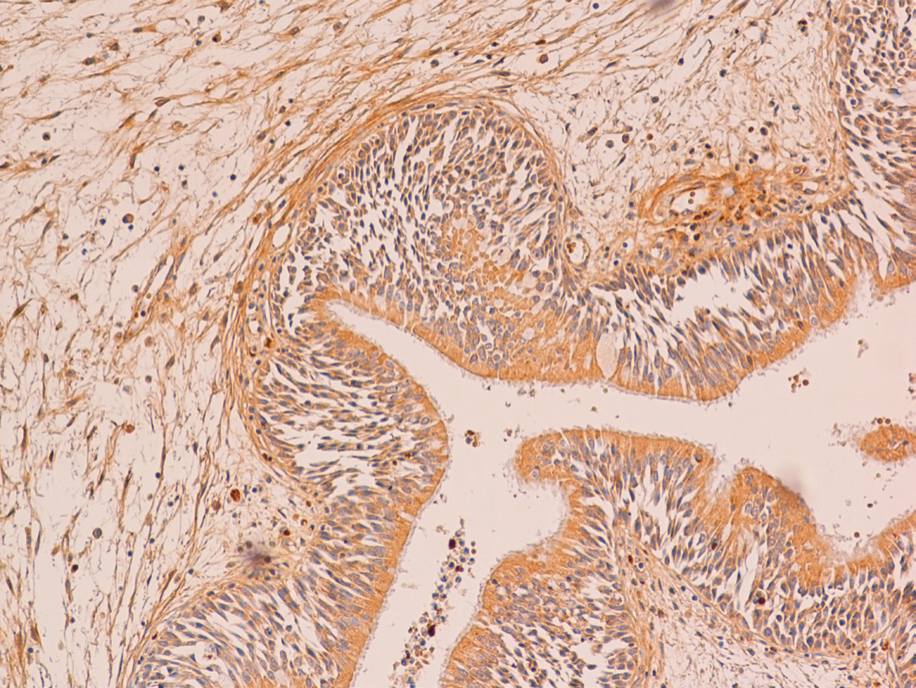
A case of relapsed IP with a low positivity for estrogen receptor (immunoperoxidase; 200×)

**Figure 5:**
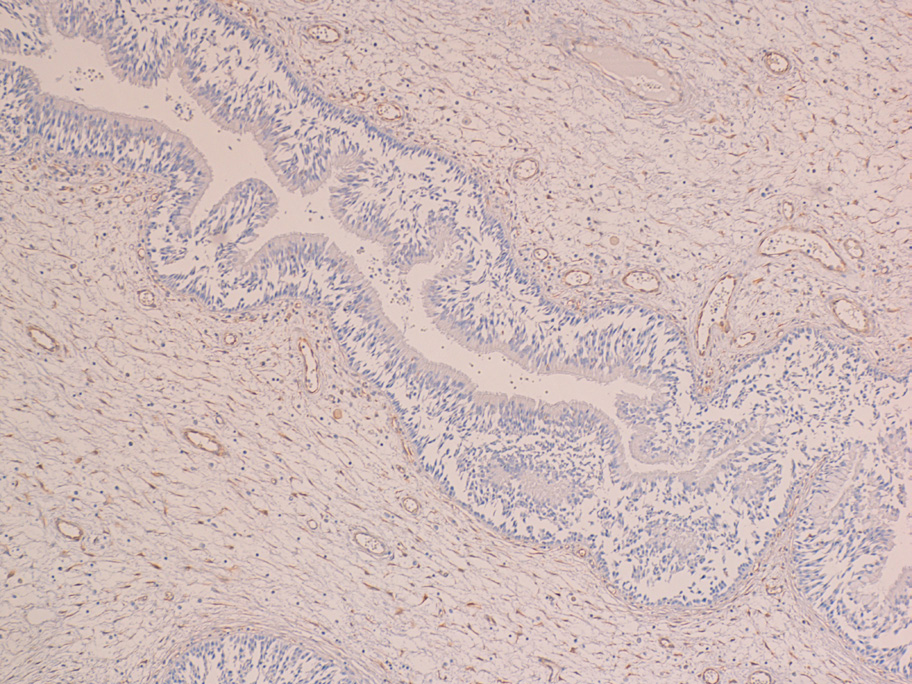
The epithelial cells of relapsed IP were negative for progesterone receptor (immunoperoxidase; 150×)

At the immunohistochemical examination, “primitive IP” showed negativity (score 0) for PGR expression in all cases (Figure 2) and low positivity (score 1) for ER in 11 cases (*P* > 0.082) (Figure 3). “Relapsed IP” showed low positivity (score 1) for ER receptors in 3 of the 21 cases (*P* > 0.068) (Figure 4), and negativity (score 0) for PGR expression in all cases (Figure 5). ER expression and sinonasal localization are summarized in Table 1. Moreover, cases of “primitive IP” with ER expression, 8 males (72.7%) vs 3 females (27.3%) (*P* < 0.044), showed a gender prevalence. In addition, 3 cases (27.2%) with ER expression showed a recurrence during follow-up (*P* > 0.068). Overall, the number of cases positive for ER expression in the “primitive IP” group, and the number of cases with ER expression in the “relapsed IP” group did not represent a statistically significant sample compared with the negative cases. All cases of “primitive IP” and “relapsed IP” showed a mean labelling index (percentage of immunostained cells) for Ki67 of 15% and no relationship between MIB-1 labelling index and estroprogestinic receptor expression was observed.

**Table 1:** Immunohistochemical analysis: number of cases/ER receptor expression

Localization	Primitive IP	Relapsed IP
Frontal sinus	10/1	2/0
Middle meatus	19/0	5/0
Maxillar sinus	16/6	6/2
Sphenoethmoidal recess	5/2	1/0
Anterior and/or posterior ethmoid	23/2	7/1
**Total (%) (*P* value)**	**11 (15%) (0.082)**	**3 (27.2%) (0.068)**

IP: Inverted Papilloma.

ER: Estrogen.

## DISCUSSION

The evaluation of expression and/or overexpression of hormone receptors in neoplastic tissues is now the fulcrum of many debates and scientific research. It seems clear that their expression implies and determines a variable degree of hormonal dependence. This principle has had a great impact in the treatment and prognosis of certain types of breast cancers. Anti-hormonal treatment with tamoxifen has provided encouraging results in controlling these tumors, also showing activity in preventing breast disease [7]. The presence of hormone receptors in other types of neoplasm has also been documented, although their clinical significance is not yet clear; and it has been documented that their expression in some cancer types does not necessarily predict the response to hormone therapy in prognostic and therapeutic approaches [8].

The expression of ER and PGR is also widely documented in the literature, even in normal and neoplastic tissues of the head and neck, but with contradictory results. Schuller et al., studied a series of 65 patients with different types of tumors in different locations of the head and neck; in this series most of the samples did not express receptors for ER and/or PGR. Only 2 out of 125 samples were considered significantly positive, the authors concluded that cancers of the head and neck should be considered hormone independent [9]. In another study Molteni et al. analyzed the expression of receptors in normal and neoplastic tissues in various sites of the head and neck. Classifying samples by anatomic origin have found significant levels of ER and PGR, particularly in normal parotid tissue and cancer, as well as in some normal and neoplastic mucosa of the floor of the mouth and nose [10].

The larynx has by far received the most attention regarding the study on sex hormone receptor expression. Ferguson et al. using immunohistochemical analysis in normal laryngeal tissues and laryngeal carcinomas, concluded that ER and PGR were both localized in the nucleus of cells and their expression was primarily in the vocal muscle in contrast to the rest of laryngeal epithelial tissue, and, in addition, no expression was found in squamous cell carcinomas of the larynx and hypopharynx [11]. Virolainen et al., documented the expression for ER and PGR in different head and neck cell lines in culture. They found significant levels of ER receptors in about 70% of laryngeal cells and in approximately 15% of cells derived from other head and neck locations. The distribution for PGR was more homogeneous, and they found that 80% of laryngeal cell lines and 55% of extralaryngeal cell lines were positive for receptors. On the basis of these results, they concluded that the hormone might be an additional treatment in the therapeutic protocol in selected cases of head and neck cancers [12].

Furthermore, Grenman et al. studied the *in vitro* effects of treatment with anti-tamoxifen in certain types of head and neck cancer, finding inhibition of growth in cell lines with significant ER and PGR expression, when these cells develop in the presence of tamoxifen citrate [7]. Based on these findings, a clinical trial by Urba et al. treated 12 patients with recurrent (on T) laryngeal squamous cell carcinomas with tamoxifen. The results of this trial, however, were discouraging, because the patients had no clinical response after treatment [13]. Another clinical trial was performed by Mattox et al. using the antiandrogen flutamide to treat 10 patients with laryngeal cancer and one patient with floor-of-the-mouth cancer with almost no results [14].

The characteristics of IP are now universally accepted: it is almost always unilateral, usually originates from the side wall of the nose, is more common in men than women (ratio of 4:l approximately), the highest incidence occurs in the sixth and seventh decades of life. Although IP is benign, it may be confused with a variety of other pathologies such as inflammatory polyps, respiratory epithelial adenomatoid hamartoma and low-grade adenocarcinomas [15–16]. The recognition of this entity is important as complete endoscopic excision is curative. The current limit of such an approach can be represented by a widespread involvement of a hyperpneumatization of frontal sinus, or lesions affecting the intracranial and infraorbital regions. In these circumstances, radical surgery can be better guaranteed by an external approach. These tumors also have a tendency to recur, are locally destructive and have significant association with squamous cell carcinoma (2% to 26% with an average of 9.2%) [17]. The significantly higher incidence in males suggests possible sexual hormone dependence. Only two studies in 1994 and 1998 studied the expression of sex hormones receptors on limited number of case histories are reported in the literature [18–19]. The analysis of these studies showed the absence of PGR-ER receptor expression or weakly positive for PR. Even if IP is about four times more common in men than in women, we obtained a comparable number of samples from both sexes to eliminate the male bias in receptor expression. After making this initial selection, the tissue samples analyzed were randomly obtained.

We found no PGR expression in the samples analyzed, except in 11 cases in the group with primary tumor and 3 cases in the group with relapsed tumor, which were weakly positive for ER (Table 1). These results obtained on a numerically significant sample and for the first time on a sample of patients with tumor recurrence, assign a role of an independent factor in the etiopathogenesis of sinonasal IP to the sex hormones examined.

## MATERIALS AND METHODS

### Study participants

In the period between 2006 and 2016, at the ENT Department of the University of Catania (Italy), in collaboration with the Section of Anatomic Pathology of the same University, a prospective study was carried out to identify the presence of receptors for estrogen (ER) and progesterone (PGR) in surgical specimens of 73 patients who had undergone sinus surgery for a first manifestation of sinonasal IP. In these patients, categorized as “primitive IP” group, for lesions limited to the middle meatus, anterior and/or posterior ethmoid sinuses and/or sphenoethmoidal recess, the endoscopic endonasal approach included an anterior and posterior ethmoidectomy, large middle meatal antrostomy, sphenoidotomy and partial and/or total middle turbinectomy. For lesions of the maxillary sinus, resection included, in addition to the previous surgical steps, also a medial maxillectomy with or without resection of the nasolacrimal duct. If the lesion involved the anterior and/or inferior posterolateral wall of the maxillary sinus an intranasal resection of the Denker type with exposure of the piriform crest and subsequent drilling of the anterior wall of the sinus was carried out. Finally, the lesions that from the anterior ethmoid involved the ostium of the frontal sinus or the small lesions occupying the frontal sinus a frontal sinusotomy sec. Draf II or III was carried out. A medial maxillectomy using external approach was performed in only 5 cases (6.8%). In all the selected cases, the precise delimitation of resection margins, the application of a subperiosteal dissection and an en bloc and/or debulking removal, guaranteed the good outcome of the surgical radicality.

The clinical and radiological follow-up was included in a range of 60–110 months, with prospective evaluation every two months during the first year and every four months thereafter. Of the 21 subjects who had developed a recurrence, categorized as “relapsed IP” group, the occurrence was within the first three years in 29% (6 cases), between 3 and 5 years in 57% (12 cases) and between 5 and 10 years in 14% (3 cases). These subjects, therefore, underwent new revision surgery and new determination of receptor expression to estrogen and progesterone.

The patient population consisted of 41 (60.2%) men and 32 (39.8%) women with an average age of 58 years (range 48–71 years) in the first group “primitive IP” and 13 (61.9%) men and 8 (38.1%) women with an average age of 65 years (range 53–78 years) in the second group “relapsed IP”. The demographic characteristics of both groups, as regards group division, gender, age and sinonasal localization are summarized in Tables 2, 3.

**Table 2:** Demographic characteristics

Groups	Primitive IP - *n* (%)	Relapsed IP – *n* (%)
**Men**	41 (56.1%)	13 (61.9%)
**Woman**	32 (43.9%)	8 (38.1)
**Age**	59 (range 48–71)	65 (range 53–78)
**Symptomatology**	Unilateral nasal obstruction (95%)	
	Serous and/or blood-serous rhinorrhea (53%)	
	Headache (27%)	
	Epistaxis (5%)	
	Facial pain (5%)	

IP: Inverted Papilloma.

**Table 3:** Sinonasal localization

Localization	Primitive IP	Relapsed IP
Frontal sinus	10 (13.7%)	2 (9.6%)
Middle meatus	19 (26%)	5 (23.8%)
Maxillar sinus	16 (22%)	6 (28.5%)
Sphenoethmoidal recess	5 (6.8%)	1 (4.8%)
Anterior and/or posterior ethmoid	23 (31.5%)	7 (33.3%)
**Total**	**73**	**21**

IP: Inverted Papilloma.

Statistical analysis was performed by using SPSS for WINDOWS (version 13.0; SPSS, Chicago, IL, USA). The results were analyzed using the non-parametric Mann-Whitney test, which was used to assess the statistical significance and differences between categories. In all cases, *P* values of < 0.05 were considered statistically significant.

### Anatomic pathology section

Four-mm serial sections from formalin-fixed, paraffin-embedded blocks of IP representative areas were cut for each case. Immunohistochemistry was then performed on the sections mounted on poly-L-lysine-coated glass slides. Deparaffinized and rehydrated sections were incubated for 30 min in 3% H_2_O_2_/methanol to quench endogenous peroxidase activity, and then rinsed for 20 min with phosphate-buffered saline (PBS) (Bio-Optica M107, Milan, Italy). Non-specific protein binding was attenuated by incubation for 30 min with 5% horse serum in PBS. Sections were then placed in a microwave oven at 750 MHz for the revelation of antigenic sites. Specimens were incubated overnight with the estrogen receptor a antibody (monoclonal mouse, clone 1D5, at a dilution of 1:35, Dako Cytomation, Glostrup, Denmark), progesterone receptor antibody (polyclonal rabbit, at a dilution of 1:50, Dako Cytomation, Glostrup, Denmark) and MIB-1, a monoclonal antibody directed against the Ki-67 antigen (1:75, Dako Corporation, Glostrup, Denmark). The antibodies were applied directly to the section and the slides were incubated overnight (4°C) in a “humidified chamber”. The sections were washed three times with PBS at room temperature. Immune complexes were subsequently treated with the secondary biotinylated antibody and then detected by streptavidin peroxidase, both incubated for 30 min at room temperature (Vectastain ABC kit, Vector Laboratories, Burlingame, CA, USA). After rinsing with 3 changes of PBS, the immunoreactivity was visualized by development for 2 min with 0.1% 3.3′-diaminobenzidine and 0.02% hydrogen peroxide (DAB substrate kit, Vector Laboratories Burlingame Calif., USA). Sections were counterstained with Mayer's hamatoxylin, mounted with permanent mounting medium and examined by light microscopy.

A semi-quantitative assessment of estrogen and progesterone receptor expression was performed assigning cases to one of the three following categories: (a) score 0, when the stained cells were from 0 to < 5% of the total; (b) score 1, when the stained cells were from > 5 to < 50% of the total cell population; (c) score 2, when the stained cells were > 50%. Positive controls consisted of tissue specimen sections of human breast tissue. A negative control was performed in all cases by substituting the primary antibody with normal mouse serum. Negative controls in all instances resulted in a negative immunoreactivity for estrogen and progesterone receptors. Only nuclear staining of epithelial cells was evaluated.

MIB-1 labeling index was evaluated in the highest immunoreactivity fields. It was expressed as a percentage and was determined by dividing the number of positive staining nuclei by 1000 tumor cells.

## CONCLUSIONS

Our results suggest that sinonasal IP is a benign tumor independent of estrogen and progesterone, and the receptors for these hormones are therefore unsuitable as predictors of relapse or possible prognostic indicators and therapeutic targets.
